# Sponge Grafting

**Published:** 1882-02-20

**Authors:** 


### SPONGE GRAFTING.

Among the new things recently introduced into medicine, "sponge grafting" stands conspicuous. The process consists, nothing more or less, than in introducing into the system a piece of sponge, which is intended to do its work as a stimulus to the repariative process, and then to be absorbed and eliminated. Dr. D. J. Hamilton, pathologist to the Edinburgh Royal Infirmary, claims the honor of having introduced the method, and in the November number of the Edinburgh Medical Journal he reports in detail his experiments, the practical conclusions to which they led him, and the crucial tests corroborative of such conclusions.

In a paper prepared some years ago, Dr. Hamilton made the statement that the vessels of granulating surface are not newly formed, but are simply the superficial capillaries of the parts which have become displaced; that they have been thrown upwards as granulation loops by the propelling action of the heart, because the restraining influences of the skin have been removed. While making observations with which to substantiate this claim, he was struck with the similarity of the process of vasculation as seen on a granulating surface, and that which occurs when a blood-clot or fibrinous exudation is replaced by vascular cicatricial tissue. Blood-clot or fibrinous lymph he came to regard as merely playing a mechanical and passive part in any situation where it becomes replaced by fibrous cicatrix, and that their vascularization is not owing to new formation of blood-vessels, but rather to a displacement and pushing inwards of the blood-vessels of surrounding tissues. Being convinced that the blood-clot, or fibrinous lymph, before organization takes place, was just so much dead matter in a tissue, it occurred to him that if some dead porous animal tissue could be substituted for the natural exudate it would, in course of time, become vascularized and replaced by cicatricial tissue. An accidental circumstance in the summer of 1880 suggested to him that in sponge we have a substance which he had long vainly sought to discover. It is porous (thus imitating the fibrinous network in a blood-clot or fibrinous lymph), it is an animal tissue, (thus, like cat gut, it is capable of absoption), and it is of such texture as permits of its adaptation to surfaces and adjustment to cavities.

Having arrived at the above conclusions by a process of reasoning, Dr. Hamilton reports five experiments in which these conclusions are strongly sustained. One of these will be sufficient here: A woman had several ulcerated wounds on different parts of her body. One of these, circular in shape, five inches in diameter, and from half to three-quarters of an inch deep, was on the outside of her leg. The edges were indurated, slightly raised, and in some places undermined. There was a cellular tissue slough at the deepest part of the wound, which gave the whole ulcer a somewhat putrefactive odor; the rest of the floor was in a granulating condition. This one was selected for experiment. August 3d, 1880, it was filled with one large piece and several small pieces of very fine sponge, which had been prepared by dissolving out the siliceous and calcareous salts by means of a dilute mineral acid (nitro-hydrochloric), subsequently washing in liquor potassa, and then finally steeping in 1 to 20 solution of carbolic acid in water. The sponge in this instance has soaked for some months in the carbolic solution, but Dr. Hamilton does not regard such prolonged soaking necessary. The sponge in the central part of the wound rose a little higher than the edges, so that at its greatest thickness it must have measured from half to three-quarters of an inch, and five inches wide. It was made to fit the wound very accurately, and was inserted beneath the undermined edges. A

piece of green protective was placed on the surface, and, above this, lint soaked in a 1 to 20 solution of carbolic acid in glycerine, with a little tincture of lavender in it. The whole was covered with a pad of boracic lint. An ordinary bandage was then applied, and the patient kept in bed with limb at absolute rest.

Without reprodueing the detailed daily report of this case, it is sufficient to say that the wound was dressed daily, such secretions as had oozed through being washed off, but the sponge left undisturbed. On January 5th, 1881, the patient was exhibited to the Medico-Chirurgical Society, and not a vestige of the sponge remained, and the wound was changed to a superficial, typical, granulating surface, measuring about 1½ inches diameter.

The first experiment convinced Dr. Hamilton that if sponge be placed over a granulating surface its interstices will, in the course of time, become filled with blood-vessels and cicatricial tissue, just as in the case of a blood-clot, and, ultimately, that the whole of the sponge will disappear in the wound, leaving an organizing mass of new tissue in its place. The vacuites in the sponge appear to be specially adapted for allowing of this, and the frame-work of keratode affords support to the young vessels which are formed within it. It further showed that even where the wound continues in a putrescent condition, organization will still go on. In the case of the blood-clot, putrefac ion tends to destroy it, in that of the sponge, its texture being more resistent, it does not seem to make much difference. There is no difficulty in keeping the sponge aseptic in a wound, provided it be so at the time of its introduction.

Dr. Hamilton's subsequent experiments were strongly corroborative of the first, and if the procedure shall prove equally successful in the hands of others, a very important device will be found to have been placed in the hands of surgeons.—[Michigan Medical News.

